# Automated Protein Subfamily Identification and Classification

**DOI:** 10.1371/journal.pcbi.0030160

**Published:** 2007-08-17

**Authors:** Duncan P Brown, Nandini Krishnamurthy, Kimmen Sjölander

**Affiliations:** Department of Bioengineering, University of California, Berkeley, California, United States of America; University of California Davis, United States of America

## Abstract

Function prediction by homology is widely used to provide preliminary functional annotations for genes for which experimental evidence of function is unavailable or limited. This approach has been shown to be prone to systematic error, including percolation of annotation errors through sequence databases. Phylogenomic analysis avoids these errors in function prediction but has been difficult to automate for high-throughput application. To address this limitation, we present a computationally efficient pipeline for phylogenomic classification of proteins. This pipeline uses the SCI-PHY (Subfamily Classification in Phylogenomics) algorithm for automatic subfamily identification, followed by subfamily hidden Markov model (HMM) construction. A simple and computationally efficient scoring scheme using family and subfamily HMMs enables classification of novel sequences to protein families and subfamilies. Sequences representing entirely novel subfamilies are differentiated from those that can be classified to subfamilies in the input training set using logistic regression. Subfamily HMM parameters are estimated using an information-sharing protocol, enabling subfamilies containing even a single sequence to benefit from conservation patterns defining the family as a whole or in related subfamilies. SCI-PHY subfamilies correspond closely to functional subtypes defined by experts and to conserved clades found by phylogenetic analysis. Extensive comparisons of subfamily and family HMM performances show that subfamily HMMs dramatically improve the separation between homologous and non-homologous proteins in sequence database searches. Subfamily HMMs also provide extremely high specificity of classification and can be used to predict entirely novel subtypes. The SCI-PHY Web server at http://phylogenomics.berkeley.edu/SCI-PHY/ allows users to upload a multiple sequence alignment for subfamily identification and subfamily HMM construction. Biologists wishing to provide their own subfamily definitions can do so. Source code is available on the Web page. The Berkeley Phylogenomics Group PhyloFacts resource contains pre-calculated subfamily predictions and subfamily HMMs for more than 40,000 protein families and domains at http://phylogenomics.berkeley.edu/phylofacts/.

## Author Summary

Predicting the function of a gene or protein (gene product) from its primary sequence is a major focus of many bioinformatics methods. In this paper, the authors present a three-stage computational pipeline for gene functional annotation in an evolutionary framework to reduce the systematic errors associated with the standard protocol (annotation transfer from predicted homologs). In the first stage, a functional hierarchy is estimated for each protein family and subfamilies are identified. In the second stage, hidden Markov models (HMMs) (a type of statistical model) are constructed for each subfamily to model both the family-defining and subfamily-specific signatures. In the third stage, subfamily HMMs are used to assign novel sequences to functional subtypes. Extensive experimental validation of these methods shows that predicted subfamilies correspond closely to functional subtypes identified by experts and to conserved clades in phylogenetic trees; that subfamily HMMs increase the separation between homologs and non-homologs in sequence database discrimination tests relative to the use of a single HMM for the family; and that specificity of classification of novel sequences to subfamilies using subfamily HMMs is near perfect (1.5% error rate when sequences are assigned to the top-scoring subfamily, and <0.5% error rate when logistic regression of scores is employed).

## Introduction

While millions of novel genes have been discovered in recent years, the *function* of the majority of genes remains unknown. Since experimental characterization of gene function is neither simple nor inexpensive, computational methods have been developed to predict function, with varying levels of accuracy [1,2]. Among the most exciting methods for predicting gene function developed in recent years is an approach called *phylogenomics* [3].

The standard protocol for functional classification of novel genes is transfer of annotation from a database hit, i.e., predicting function based on sequence similarity between an unknown gene and one whose function is (presumably) known. This concept has given rise to many functional annotation methods [4–9]. Unfortunately, transferring the annotation of the highest-scoring database hit has been shown to be prone to systematic error caused by changes in protein function following gene duplication, speciation, domain shuffling, and other evolutionary processes [10–13]. Moreover, existing database annotation errors can be propagated by this approach [14].

By contrast, phylogenomic inference employs phylogenetic analysis of an entire protein family in order to predict function for individual members. By overlaying experimental data on the phylogenetic tree, a biologist can identify where, in evolution, genes may have been duplicated, lost, or transferred horizontally, or adopted new functions. Phylogenomic analysis thereby enables a biologist to “fill in the blanks” with an extremely low error rate, and often with significant detail [15–18]. The value of phylogenomic approaches to gene function prediction has spurred the recent development of a number of methods automating one or more steps in the phylogenomic inference protocol. Many of these automate the process of collecting, aligning, and clustering homologs, but do not actually assign function [19–24]. Others focus primarily on the assignment of function [25–29]. While each of these approaches has distinct advantages, none are designed for fast classification of novel sequences or identification of new functional subtypes. The protocol outlined in this paper enables improved specificity of functional inference and facilitates prediction of functional shifts in new sequences.

We present two methods useful in automating phylogenomic inference: de novo subfamily identification using SCI-PHY (Subfamily Classification in Phylogenomics) and classification of novel sequences using subfamily hidden Markov models (HMMs). These two methods form part of a computational pipeline for phylogenomic inference of gene function that was originally developed for the functional classification of the human genome at Celera Genomics [30]; the methods reported here have been revised to improve both accuracy and computational efficiency. In the first stage, functional subfamilies are defined either through the use of expert knowledge or computationally. In the second stage, these subfamilies are used to construct subfamily Hidden Markov models (SHMMs) [31]. In the third stage, sequences predicted to be members of the family are then tested for subfamily membership and either assigned to one of the existing subfamilies or predicted to represent an entirely novel subtype not included in the original dataset.

### De Novo Subfamily Identification

De novo subfamily identification—partitioning of sequences in a dataset into subtypes—provides two advantages for high-throughput systems of functional classification. First, assuming at least one subfamily member has been experimentally characterized, it becomes possible to infer function for other members of the subfamily. Second, the identification and curation of known subfamilies enables biologists to use sequence-based classification methods (e.g., using profiles, HMMs [32], or support vector machines (SVMs) [33]) to assign novel sequences to existing subtypes.

Existing methods for de novo identification of specific subtypes fall into two camps: those that define clusters using pairwise similarity, e.g., InParanoid [24], OrthoMCL [21], Ncut [25], CD-HIT [22,34], and those that cluster by cutting a phylogenetic or hierarchical tree, e.g., RIO [29], Orthostrapper [28], Secator [35], SCI-PHY.

Both Secator [35] and SCI-PHY identify subfamilies using hierarchical tree construction and analysis. Secator uses a sequence dissimilarity measure to define an optimal cut of the tree. SCI-PHY uses minimum description length principles from information theory to cut the tree into subfamilies [36]. SCI-PHY exploits two powerful tools to construct a hierarchical tree: Dirichlet mixture densities [37] and relative entropy [38]. Dirichlet mixture densities are used to construct profiles for subtrees due to their utility in enhancing sensitivity with no reduction in specificity [39]. Relative entropy is used as a distance function between subtree profiles to determine the join order in the tree. See Methods for details.

SCI-PHY is a fast method of subfamily identification which uses only sequence information, in contrast to phylogenetic tree methods that require species information to resolve orthologs from paralogs for functional analysis. Therefore, SCI-PHY is especially advantageous in situations where species information is not known, such as in environmental sequences. Our experiments show that SCI-PHY subfamilies correspond closely to subtypes found by experts and also to conserved clades identified using standard phylogenetic tree analysis.

### Functional Classification of Unknown Sequences to Defined Subfamilies

The availability of subfamily classifications enables high-throughput functional annotation: as new sequences are released to the sequence databases, sequence-based classification methods can be used to efficiently assign unknown sequences to pre-defined subtypes. Several classes of methods have been developed for this task. Profiles and HMMs are statistical models that generalize the information in a multiple sequence alignment (MSA) [32], and can be used to develop subfamily profiles or HMMs, as described in this paper. However, most profile/HMM libraries (e.g., PFAM [40], the NCBI CDD [41], SMART [42], etc.) have focused on modeling large diverse clusters of proteins spanning many different functions, enabling high sensitivity, but affording only a fairly coarse level of functional annotation [29].

Methods designed specifically for classification of sequences to predefined subfamilies include the profile-based method of Hannenhalli and Russell [43] and SVMs [44]. Hannenhalli and Russell developed a profile-based subfamily classification system that attempts to determine which alignment positions discriminate between subfamilies. SVMs use both positive and negative training examples to allow classification of sequences to different subtypes (e.g., [33]). Weston et al. developed a semi-supervised algorithm that incorporates unlabeled proteins into an SVM-based discriminative classifier [45].

Our approach to classification of novel sequences to functional subfamilies uses subfamily hidden Markov models and a computationally efficient scoring system [31]. Note that subfamilies may be either automatically or manually defined; the system is independent of the origin of the classification. Subfamily HMMs are constructed using an information-sharing protocol that enables small subfamilies to benefit from the information contained in the rest of the family (such as catalytic residues showing universal conservation) while retaining specificity at subfamily-defining regions or motifs. This improves the sensitivity of the subfamily HMM to detect new members while providing for extremely high specificity of classification. A preliminary study of SHMM performance on a small dataset of nine protein families has been previously published [31]. Here, we present results on a larger, representative dataset of 515 families, and compare SHMMs to other sequence classification systems.

### Identification of Novel Subtypes

The ability to predict novel subtypes in a protein family is extremely valuable in identifying functional shifts in newly sequenced genomes. In addition to classification of novel sequences to predefined subfamilies, we present a method of logistic regression of positive and negative examples for subfamilies. This method enables discrimination between novel sequences that can be reliably classified to an existing subfamily and those that are more likely to represent entirely different subtypes from any previously observed.

## Results

### Benchmark Datasets

#### SCOP-PFAM515.

515 full alignments were selected from the PFAM resource [40] to assess remote homolog detection capabilities of subfamily HMMs relative to family HMMs. We used the Structural Classification of Proteins (SCOP) resource [46] to select PFAM families that corresponded to a single SCOP superfamily, and filtered this set to choose a single representative for each SCOP fold. The dataset selected is thus not biased toward any particular fold type, and results can be expected to generalize well to novel protein families and folds.

#### Enzyme classification.

A subset of 57 families from SCOP-PFAM515 contains sequences with multiple enzymatic functions based on all four fields of their Enzyme Classification (EC) [47] number. In each of these PFAM families, only a fraction of the sequences have been assigned an EC number. The subfamily prediction methods are given all sequences to classify, but only scored on those sequences with EC numbers. To reduce the possibility that an EC number assigned to a sequence was based on a region not included in the PFAM full MSA, we ignored any members whose PFAM alignments reflected less than 75% of the sequence length.

#### EXPERT.

This dataset contains five extensively curated protein families from three different resources, with additional subdivisions of two of the families to create a total of eight classifications of expert-defined subtypes. We selected the enolase and crotonase enzyme families from the Structure-Function Linkage Database (SFLD) [48], a manually curated resource that incorporates mechanistic, sequence, and structural information for several diverse enzyme families to derive functional subtypes based on conserved chemical mechanisms. We selected the aminergic and secretin families of 7TM receptors from the GPCRDB (G-protein coupled receptor database) [49] and the nuclear hormone receptor (NHR) family from NucleaRDB [50]. The GPCRDB and NucleaRDB classify sequences at varying levels of functional specificity, indicated here by the use of level indices. For instance, NHRs have been divided into three levels, from fairly coarse (NHR level 1) to highly specific (NHR level 3). The amine receptors were subdivided by the GPCRDB into a coarser level 1 and a more specific level 2. This produced a total of eight datasets spanning different levels of functional specificity: Enolase, Crotonase, Amine levels 1 and 2, NHR levels 1–3, and Secretin.

### Results of De Novo Subfamily Identification on Benchmark Datasets

We compared SCI-PHY to three other methods for protein subfamily identification that depend only on sequence information: Secator, Ncut, and CD-HIT. CD-HIT takes a user-specified minimum percent identity as a parameter for determining cluster membership; we present results for two identity cutoffs: a comparatively low value (40%) in order to identify fairly general functional groups, and a higher value (70%) that has been identified as the minimal identity required to guarantee functional similarity within subfamilies [51,52]. We refer to these as CD-HIT40 and CD-HIT70. Results for additional percent identity values are available in Dataset S1. Secator, CD-HIT40, CD-HIT70, and SCI-PHY were compared on the EXPERT and EC datasets, spanning a total of 62 distinct protein superfamilies each containing multiple subtypes. Due to Ncut's high computational cost, we analyzed Ncut performance on the EXPERT dataset only.

We used three scoring functions—purity, edit, and variation of information—to measure the agreement between the reference subtypes in each benchmark dataset and the subfamilies predicted by the methods tested. The *purity* score is a simple measure of each method's ability to properly separate reference subtypes, measured by the fraction of predicted subfamilies that contain sequences of only one reference subtype. Since perfect purity can be achieved trivially by placing every sequence in its own class, we exclude singleton subfamilies from the purity calculation. The *edit* distance between the two dataset partitions measures the number of split or merge operations required to transform one partition into the other. For instance, if one partition contains two clusters whose members are found as one large cluster in the other partition, a single merge or split operation suffices to transform one into the other, producing an edit distance of 1. On the other hand, if a cluster of *k* members in the reference (trusted) partition is divided into *k* singletons in the predicted partition, ⌈log_2_ *k*⌉ merge operations are required. Thus, the edit distance penalizes over-division of a reference subtype more than it does two or more reference subtypes being merged. The *Variation of Information* (VI) distance [53] calculates the amount of information (in bits) within each partition that is not present in the other. A perfect score of zero indicates that the partitions are identical. The purity score and edit and VI distances were chosen to be complementary measures: purity represents the overall precision or specificity in separating functional subtypes, while the edit and VI distance are somewhat analogous to sensitivity or recall. An ideal subfamily classification will produce classes having sequences of only one type (perfect precision) and maximize the size of these clusters (perfect recall). The purity function provides a means to measure the first attribute, while the edit and VI distances provide a means to measure the second.

Finally, we also assessed agreement between SCI-PHY subfamilies and phylogenetic trees and found that SCI-PHY subfamilies typically correspond to well-supported clades within the family (Dataset S2).

#### EXPERT dataset.

These experiments highlighted the classic tradeoff between specificity and sensitivity. High purity and low edit and VI distances for a method indicate that the subfamily decomposition achieved by that method is very similar to the reference partition. Of all the methods tested, SCI-PHY has the most consistent performance in combining both high purity and low distance to the reference partition. For instance, SCI-PHY has the best (lowest) edit and VI distances of all methods tested, ranking first for four out of eight EXPERT datasets. Secator comes next, with the best edit or VI distance for two of the eight datasets. Other methods tested have either high purity but a large distance (e.g., CD-HIT70) or sacrifice purity for a lower distance (CD-HIT40 and Secator). The performance of the Ncut method differed between the families; for example, it performed very well with the crotonase family, but then clustered 317 of the 328 sequences in the aminergic family, spanning multiple GPCR subtypes into one subfamily. The single notable exception is the enolase family, for which SCI-PHY, Secator, and Ncut produced a large number of singleton clusters (52, 29, and 35, respectively), giving each a poor distance score. A detailed comparison of performance of the different methods on the adreno-receptor family of GPCRs is given in Dataset S3. An overview of all method performances on the EXPERT dataset is shown in Table 1.

**Table 1:**
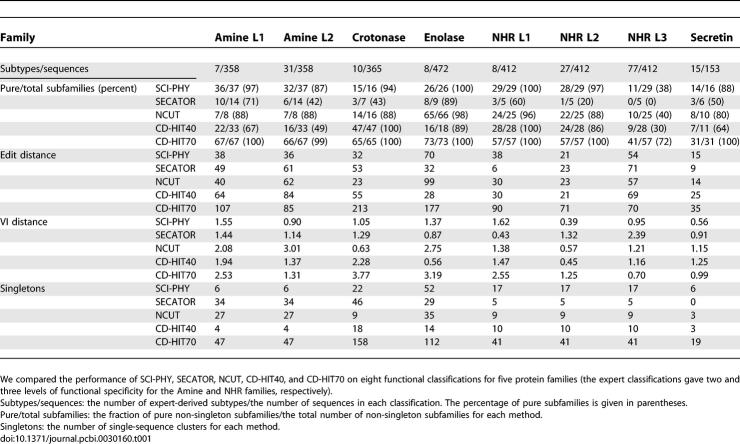
De Novo Subfamily Identification for the EXPERT Set

Assessing de novo subfamily identification accuracy by comparison with expert-defined subtypes presents unique challenges. First, there is a wide variation in expert definitions of functional classes—some expert-defined subtypes are highly specific and span short evolutionary distances, while others cluster proteins at a much coarser level and may include highly divergent sequence pairs. Similarly, subfamily prediction methods tend to aim at different points in this spectrum. For instance, CD-HIT40 clusters at a fairly coarse level and has comparatively poor purity scores for highly specific expert-defined levels, but fairly good edit and VI distance scores. By contrast, CD-HIT70′s subfamily purity is the best of the five methods tested, but it has the worst edit distance on seven out of eight classifications, and the worst VI distance on four of the eight datasets.

Comparing de novo subfamily identification methods against an expert-defined hierarchy reveals the inherent biases of these methods, as each tends to target a different level of a functional hierarchy. This is illustrated by comparison of subfamily prediction methods to the different levels in the aminergic GPCR and NHR families in the EXPERT dataset. Here, Secator's division of the NHR family is closest to the coarse level 1 classification (although Secator's purity scores are poor even at this level), while SCI-PHY and CD-HIT40 match the more specific level 2 classification. CD-HIT70 performs very well at the most specific level (NHR L3), with high purity and low VI distance. Interestingly, CD-HIT with a 50% identity cutoff (CD-HIT50) seems to give a better balance of purity and distance than CD-HIT70 (Dataset S1), despite the identification of 70% as the minimal identity required for functional specificity [51,52]. Nonetheless, CD-HIT50 has worse edit and VI distance scores than SCI-PHY in seven of the eight classifications, and only marginally better purity.

The different scoring functions used to evaluate subfamily identification highlight the standard problem in function prediction: achieving a balance between sensitivity and specificity. The purity score measures specificity, whereas the distance functions correspond more closely to sensitivity. There are subtle differences between the two distance functions. Both the edit distance and the VI distance penalize over-division as well as mixing of subtypes, but the edit distance penalizes over-division of subtypes proportionately more than joining a few subtypes into large clusters. The edit distance thereby favors methods such as Secator and CD-HIT40 that produce fairly coarse clusterings. The VI distance takes cluster size into account, and errors in large clusters (affecting many sequences) contribute more to the distance than errors in small clusters. These effects are illustrated by the change in distance-based rank between SCI-PHY and Secator for the Secretin family. On this family, SCI-PHY had a better VI distance than Secator, but a worse edit distance. Examining the two predicted partitions relative to the expert division into subtypes shows why. The SCI-PHY subfamily prediction had high purity (only one SCI-PHY subfamily merged two different subtypes together), but somewhat over-divided expert subtypes, splitting three expert subtypes into multiple subfamilies and producing six singleton subfamilies. In contrast, Secator had low purity (three of the six subfamilies produced by Secator joined several subtypes together, placing nearly 70% of the sequences in the family into mixed subfamilies) but did not subdivide expert subtypes, and very few split operations were required to obtain the expert classification.

#### Results on the EC dataset.

Performance for the EC dataset (Table 2) was quite similar to that on the EXPERT set, except that SCI-PHY has slightly lower purity than CD-HIT40 (while still maintaining superior edit and VI distances), and slightly higher distance scores than Secator, but with superior purity. On this dataset, Secator has the lowest purity over the families tested (71%), but has the best average VI and edit distance scores (0.91 and 8.9, respectively). CD-HIT70 has near-perfect subfamily purity (97%), but the worst distance scores (2.07 and 30.0). SCI-PHY lies between these two extremes: very good subfamily purity (80%) and quite close to the expert partition (1.07 and 10.8). Results for additional parameterizations of CD-HIT are given in Dataset S2.

**Table 2:**
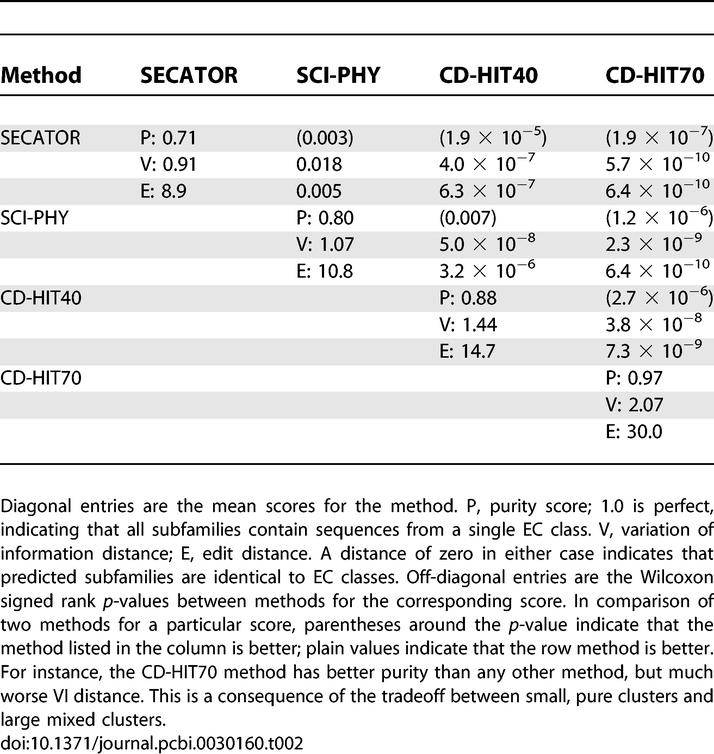
Wilcoxon Signed Rank Tests for De Novo Subfamily Detection on the EC Dataset

#### Classifying unknown sequences: Subfamily HMM classification accuracy.

HMMs and profiles are very effective at detecting distant homologies. Since primary sequence diverges more rapidly than 3-D structure, the scientific community uses the ASTRAL datasets of solved structural domains from the SCOP database [54–56] to assess performance at remote homolog detection. SCOP domain pairs belonging to the same superfamily are considered homologous. Domain pairs belonging to different SCOP folds are considered unrelated. Perfect performance requires ranking all superfamily pairs above all fold-disagreement pairs.

**Figure 1:**
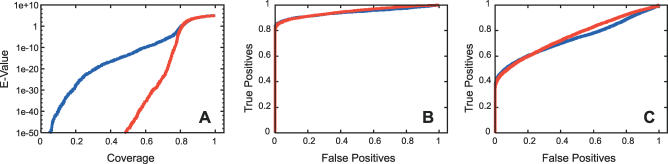
Comparison of Family and Subfamily HMM Performance on Remote Homolog Detection Blue: family HMM results. Red: subfamily HMM results. (A) Coverage (*x*-axis) is plotted against e-value (*y*-axis). Coverage (or recall) is the fraction of homologous pairs (i.e., from the same SCOP superfamily) that receive a score of equal or greater significance. The e-value curves converge at a coverage of 0.79, the same coverage at which false positives first arise. This corresponds to an e-value of approximately 0.01. (B) ROC curve for family and subfamily HMMs, weighted by superfamily size. Subfamily HMMs receive an AUC of 0.947; family HMMs receive 0.943. (C) ROC curve for unweighted data. Subfamily HMMs and family HMMs have AUCs of 0.758 and 0.740, respectively. Together, these data show that while subfamily HMMs do not detect more homologs at a given false positive rate, they do find many more homologs at a given significance cutoff.

We compared the ability of family and subfamily HMMs (for subfamilies defined by SCI-PHY) to identify remote homologs using the SCOP-PFAM515 dataset, spanning 515 PFAM families representing unique 3-D folds. Results of these experiments show that subfamily HMMs significantly increase the separation between true homologs and spurious matches by improving the scores of related sequences. For instance, at an e-value cutoff of 10^−20^, SHMMs detected 73% of SCOP superfamily members, whereas family HMMs detected only 31%. This increase in score significance for homologous sequences comes at no cost in error rate: ROC plots of subfamily and family HMMs superpose closely, with the AUC of subfamily HMMs being slightly greater than the AUC of family HMMs (Figure 1). Our information-sharing protocol improves the overall sensitivity of subfamily HMMs: comparisons of the method with “naïve” subfamily HMMs—those built directly from subfamily alignments without information sharing—shows that, on average, “informed” SHMMs improve e-values for family members by approximately nine orders of magnitude (Dataset S4).

We tested subfamily classification accuracy using leave-one-out experiments. Ten sequences from each family in the SCOP-PFAM515 dataset were individually removed from the alignment, SHMM parameters were estimated without the withheld sequence, and the sequence was then scored against all SHMMs. 98.5% were assigned to their original subfamily, producing an error rate of only 1.5%. Since many of the sequences tested were highly similar to sequences present in the alignments, we also tested classification accuracy following alignment editing to remove sequences with different levels of percent identity to the chosen sequence. We compared SHMM performance with the use of BLAST, and to the sub-profile method of Hannenhalli and Russell [43]. The results are given in Table 4. All three methods achieve similar performance on this dataset. We also assessed subfamily HMM and Hannenhalli and Russell sub-profile performance based on subtypes defined by experts rather than by SCI-PHY, and found similar results (Dataset S5).

**Table 3:**
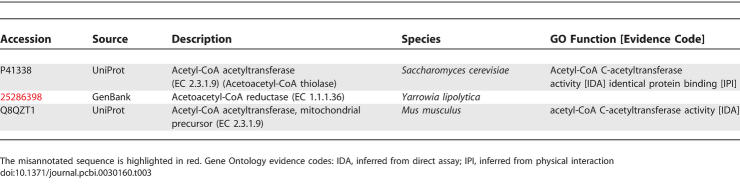
Error in Function Prediction Is Revealed by Clustering the Misannotated Sequence with Its Homologs Using SCI-PHY

**Table 4:**
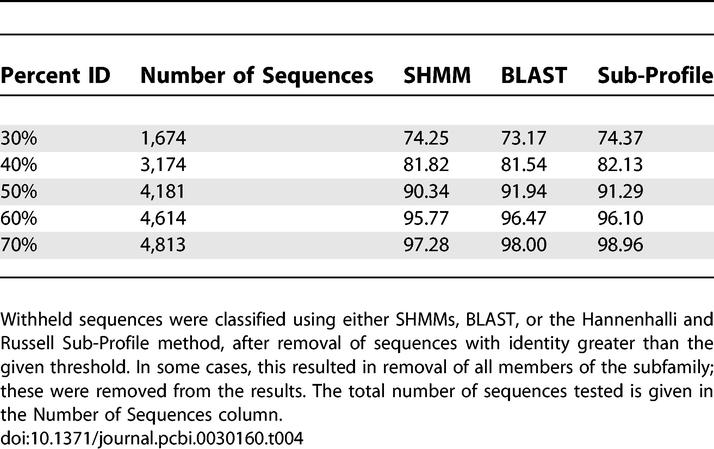
Novel Sequence Classification on the SCOP-PFAM515 Dataset, after Removal of Sequences Similar to the Target Sequence

In these experiments, the Hannenhalli and Russell sub-profile method showed only a marginal improvement in classification accuracy over the BLAST and HMM methods, in contrast to earlier work, which showed a dramatic improvement over BLAST and HMM-based classification for more divergently related families [43]. We believe that this result is due to the coarse classification used in the previous experiments, in which highly diverse families were divided into only two or three subtypes which were individually quite diverse. The sub-profile method attempts to determine which columns discriminate between subtypes, but having a larger number of subtypes reduces the likelihood that any column will show distinct conservation patterns in *all* the subtypes. More specific partitions thus reduce the advantage of the sub-profile method. The marginal improvement of the sub-profile method to SHMMs and BLAST in these experiments may also be related to our providing it the “true” alignment from the input MSA, instead of requiring it to align the sequences separately, as was required by the SHMM and BLAST methods.

#### Classifying unknown sequences: Detecting novel subfamilies.

Protein families naturally expand in size to accommodate additional homologs produced by genome-sequencing initiatives. Many of these new members will belong to known subtypes, but some will represent novel subtypes having distinct functions. We have developed an online algorithm to assess the likelihood that an unknown sequence represents a novel subfamily.

**Figure 2:**
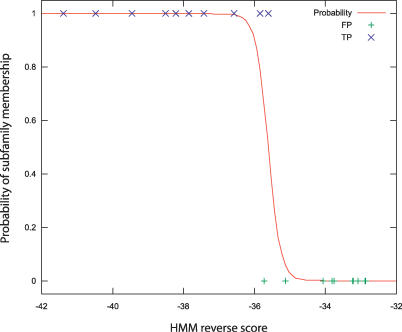
Logistic Regression for Novel Subtype Identification The logistic regression fit for an example subfamily is shown. True subfamily members (X) and other family members (+) are shown, together with the fitted curve. When the two classes cannot be completely separated, as in this case, we see a smooth transition in the probability of subfamily membership.

Since classification of sequences to existing subfamilies based on top subfamily HMM scores has an extremely low error rate, we treated this task as a binary classification problem, asking the question, “Does the test sequence belong to the top-scoring subfamily, or does it represent a novel subtype?” We used logistic regression to predict the probability of subfamily membership based on the HMM reverse score (Figure 2). The sigmoid logistic curve gives a smooth transition between obvious members and obvious non-members, and gives a better sense of the confidence of our prediction than a simple score threshold. At the same time, it is easy to fit and makes no assumptions about the distributions of the scores.

**Figure 3:**
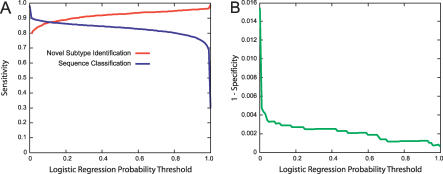
Novel Subtype Identification and Classification Accuracy as a Function of the Threshold on Subfamily Membership Probability (A) The red line shows the fraction of novel subfamilies correctly detected; the blue line shows the fraction of subfamily members correctly classified in leave-one-out experiments. Novelty detection is quite robust to the threshold setting, obtaining 80% success rate even at the lowest threshold (0.01). (B) The fraction of sequences classified to an incorrect subfamily during leave-one-out experiments. While low to begin with, the false positive error drops dramatically with the imposition of even a small threshold. A threshold of 0.10 probability of subfamily membership seems to be optimal; the false-positive classification rate is just 0.3%, while overall subfamily classification and novel subtype detection accuracy are both 88%. The *x*-axis shows the logistic regression probability threshold for subfamily membership assignment.

We tested novel subtype identification as follows. For each PFAM MSA containing at least three SCI-PHY subfamilies, we removed an entire SCI-PHY subfamily (selected at random) and re-estimated HMM parameters for the remaining subfamilies. Retained sequences were used to fit regression curves for each SHMM by all-against-all scoring within the family. Each sequence from the withheld subfamily was then scored against the new set of SHMMs, and the probability that it belonged to the top-scoring subfamily was calculated. We assessed subtype detection sensitivity for a range of membership probability thresholds (Figure 3A). Even at very low acceptance thresholds, logistic regression correctly discriminates between novel sequences and members of existing subfamilies; at the lowest tested threshold of 0.01, 80% of the sequences were correctly classified as novel subtypes.

**Figure 4:**
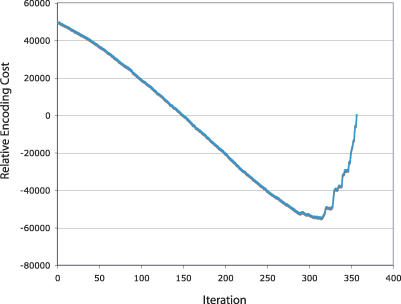
The Encoding Cost as a Function of the SCI-PHY Iteration for the Secretin Family We subtract the encoding cost of the null hypothesis (that all sequences belong in a single subfamily) from the cost of encoding the subclass alignments at each iteration of the algorithm (*y*-axis: Cost_iteration_ − Cost_null_). At program commencement, the number of subclasses equals the number of sequences and the encoding cost is high. The encoding cost curve decreases steadily to a minimum when similar sequences are joined and then increases as subtrees with different amino acid preferences are joined. The point in the agglomeration for which the encoding cost is minimal is used to determine a cut of the tree into subtrees, defining the SCI-PHY subfamily decomposition. If the minimum occurs when the encoding cost is zero, then all sequences are placed in a single class (i.e., no subfamilies are predicted). Negative “Encoding Cost” values indicate savings relative to the null hypothesis, and provide support for a division of the sequences into two or more subfamilies.

We then assessed the impact of this classification protocol for the complementary task: subfamily classification of a test sequence belonging to an existing subfamily. In this case, we repeated the 5,103 leave-one-out experiments described in the previous section, this time fitting regression curves to each subfamily and calculating the probability that the withdrawn sequence was a true member of the best-scoring subfamily. At high stringency (i.e., requiring a high subfamily membership probability), the number of mis-classified sequences (false positives) is minimal, but sensitivity is reduced (Figure 3A). At a threshold of 0.10, both classification accuracy and novel subtype detection algorithms produce an impressive success rate of 88%.

An unanticipated effect of this thresholding process was to greatly reduce the fraction of false positive classifications (Figure 3B). Virtually all classification errors in the leave-one-out experiments possessed scores well below the norm for their top-scoring subfamily, and were eliminated at very low thresholds. For instance, a membership probability threshold of 0.05 lowered the error rate from 1.5% to 0.3%.

**Figure 5:**
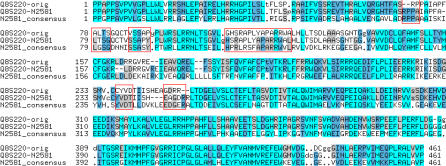
Discordance between Subfamily Membership and Top-Scoring SHMM Can Be Indicative of Misalignments Sequence Q8S220, a singleton subfamily, was classified to its sibling subfamily, N2581. We show a comparison of the sequence as aligned in the original MSA (Q8S220-orig) and after alignment to SHMM N2581 (Q8S220-N2581). The consensus sequence for SHMM N2581 is also shown (N2581-consensus). After realignment, much of the sequence has been shifted, and several motifs now clearly match the N2581 consensus sequence (red boxes).

*Detecting errors in the input alignment using SHMMs:* based on prior observations that substantial disagreements between a SCI-PHY subfamily decomposition and classification based on top-scoring SHMM are often diagnostic of errors in the input MSA, we examined errors in classification in the first set of novel subtype detection experiments. We found that many of these errors were singleton subfamilies whose sequences were assigned incorrectly to another subfamily. We suspected that each of these sequences may have been misaligned in the original MSA, causing SCI-PHY to classify the sequence as a separate subfamily instead of joining it to the top-scoring subfamily. To test this hypothesis, we selected all singleton sequences that were erroneously accepted by another subfamily at a probability threshold of 0.7, and we re-aligned each to its top-scoring SHMM. 13 of the 47 tested singleton sequences showed substantial changes in alignment between the initial input MSA and the new alignment (≥5 residues shifted). When these revised alignments were submitted to SCI-PHY, six of the sequences were joined into the (now larger) subfamily to which they had been assigned in the novel subtype detection experiments. We show an example in Figure 5.

#### Genome-scale precomputation of subfamilies and subfamily HMMs.

We have applied the methods described here in the construction of a phylogenomic HMM library, the PhyloFacts Universal Proteome Explorer, with more than 40,000 protein family “books” and more than 1.2 million HMMs to enable subfamily classification of novel sequences [57]. Each book contains an MSA, SCI-PHY subfamily decompositions, one or more phylogenetic trees, family and subfamily hidden Markov models, experimental data, Gene Ontology annotations, predicted 3-D structures, PFAM domains, and more. We also provide the SCI-PHY Web server that allows users to upload an MSA for subfamily identification and subfamily HMM construction [58]. Biologists wishing to provide their own subfamily definitions can do so. See http://phylogenomics.berkeley.edu/SCI-PHY/.

#### Annotation transfer and detection of misannotated sequences.

We present an illustration in which clustering sequences using SCI-PHY enables detection of existing errors in database annotations.

The GenBank sequence JC7675 (Table 3, shown in red) from yeast has been annotated as “Aceto-acetyl-CoA reductase (EC 1.1.1.36).” However, the gene coding for this protein, *pat1,* has been shown to be a peroxisomal acetoacetylCoA thiolase (EC 2.3.1.9) required for n-decane utilization in Yarrowia lipolytica [59]. The Thiolase family includes both acetoacetylCoA transferase (EC 2.3.1.9) and 3-ketoacylCoA thiolase (EC 2.3.1.16). The SCI-PHY subfamily classification for this sequence reveals that it is more closely related to EC 2.3.1.9, encoding “AcetylCoA-acetyl transferase.” Aceto-acetyl-CoA reductase catalyzes redox reactions, while acetylCoA-acetyl transferase performs a thiolytic cleavage. The clustering of proteins into SCI-PHY subfamilies helps detect and correct this possible misannotation. Note that PFAM is unclear on the classification of this sequence, placing it into the thiolase family which includes both EC 2.3.1.9 and EC 2.3.1.16. Detailed phylogenomic analysis for this protein can be found in the PhyloFacts book “Thiolase” (http://phylogenomics.berkeley.edu/book/book_info.php?book=bpg000891).

Additional examples of effective annotation transfer and error detection are given in Dataset S6.

## Discussion

Phylogenomic analysis is widely regarded as the method of choice for high-accuracy functional annotation but has had limited application due to the technical complexity of this protocol. This paper focuses on methods to automate a phylogenomic pipeline using defined subfamilies followed by construction of HMMs for these subfamilies, which can then be used to classify novel sequences.

A large fraction of phylogenomic inference tools focus on the identification of orthologs as the basis of annotation transfer, under the assumption that orthologs—related by speciation from a common ancestor—are likely to maintain the same function. Methods developed for phylogenomic inference that use species information in conjunction with phylogenetic tree analysis to identify orthologs include RIO [29], Orthostrapper [28], and SIFTER [27]. While functional inference based on orthology is likely to have the highest specificity, this requirement is effectively quite limiting in practical application. First, annotation transfer based on strict orthology to characterized sequences will be highly restricted due to the small fraction of sequences having experimentally verified function (see, e.g., [27]). Second, orthology detection requires species information; this is not always available, for example, in environmental sequence analysis [60,61]. For these reasons, methods of subfamily identification that use only sequence information (such as SCI-PHY) can be quite useful. In addition, restricting functional inferences to strictly defined orthologs may be unnecessarily restrictive as sub-functionalization following gene duplication is quite common: our analyses of the five families included in the EXPERT dataset show that a significant fraction of expert-defined subtypes include paralogous sequences.

Any division of a family into functional subtypes is somewhat arbitrary, as proteins have different levels of molecular function ranging from the fairly coarse (e.g., catalytic activity) to highly specific (e.g., substrate recognition). For this reason, some protein classification databases, such as the GPCRDB [49], present a hierarchy of functional classes rather than a single subfamily definition. For instance, the human type 1 serotonin receptor (UniProt accession P08908) is classified to the GPCRDB serotonin type I receptors, a subtype of the serotonin receptors, which belong to the amine (or aminergic) GPCR family, which forms a subclass of Class A rhodopsin-like GPCRs. Other resources provide single-level classifications, which similarly range from coarse to highly specific. Each of these approaches to functional classification has its own merits. Similarly, biologists can select subfamily prediction methods appropriate to an intended use: for highly specific definitions of molecular function, CD-HIT70 and SCI-PHY will be most appropriate. For more coarsely grained clusters, CD-HIT40 and Secator will be preferred.

Subfamily identification methods that rely on an MSA as input, including SCI-PHY and Secator (and most phylogenetic tree construction algorithms), tend to be quite sensitive to alignment errors. We therefore recommend careful attention to the construction of the MSA for the family. Removing columns having many gap characters is analogous to alignment masking prior to phylogenetic tree construction, and is recommended. A protocol for collecting and aligning homologs is given in [18].

### Using Subfamily HMMs to Classify Novel Sequences

Our results show that subfamily HMMs provide high specificity of sequence classification to functional subtypes, providing a kind of automated phylogenomic inference that approximates the results achievable from a more compute-intensive phylogenetic reconstruction. The information-sharing protocol we present produces subfamily HMMs that generalize effectively to distant homologs. Information sharing leverages available training data and helps to smooth estimated amino acid distributions to prevent overly specific HMM parameters in small subfamilies. This information-sharing protocol more efficiently separates homologs from non-homologs than subfamily HMMs without information sharing, but at a slight cost in subfamily specificity (i.e., the error rate for subfamily classification without information sharing is 0.8%, while our standard information-sharing protocol has an error rate of 1.5%).

In these experiments, family and subfamily HMMs showed similar classification error rates, although subfamily HMMs produce much more significant e-values for true positives, in addition to identifying subfamily membership. This suggests a simple way to reduce the computational burden of using SHMMs, which we use in practice. Rather than scoring novel sequences against all SHMMs from all families, we screen sequences for family membership using family HMMs and then identify the appropriate subfamily by scoring the sequence only to the SHMMs of that family. Since most HMM libraries contain thousands of families, the average increase in scoring runs due to the use of SHMMs is then marginal.

Logistic regression of subfamily HMM scores enables us to discriminate between sequences representing entirely novel subtypes and sequences that can be assigned to existing subtypes. This confers a unique capability to subfamily classification systems that is critical to prevent overly specific (incorrect) predictions of molecular function for novel sequences.

All methods of constructing subfamily models as a means of classifying novel sequences will be sensitive to the inclusion of outlier sequences in a family. A single or small number of outlier sequences normally have minimal effect on a profile or HMM constructed for the family as a whole (since their contribution is typically washed out by the dominant group) and may remain undetected. However, the use of subfamily models, whether through subfamily HMMs, as outlined here, or by another method, can magnify the power of these outliers to attract and recruit their relatives. This may be desirable when outliers are actual homologs, but is generally not desirable in the case of spurious database hits. However, if non-homologous outliers can be flagged, their corresponding subfamily models can be used as decoys, differentiating true family members from those that only appear to be related.

## Materials and Methods

### SCI-PHY de novo subfamily identification.

The input to SCI-PHY is an MSA, from which a hierarchical tree and subfamily decomposition are estimated. SCI-PHY uses agglomerative (bottom-up) clustering to construct a hierarchical tree: the input objects form the leaves in the tree; similar objects are joined by edges to form subtrees, and the process is iterated until a rooted tree is obtained.

**Algorithm**

*Input:* MSA

*Initialization:*

Each sequence forms a separate class (leaf in tree). For each class, construct a profile, using Dirichlet mixture densities [37]. Compute the pairwise distances between all classes, using relative entropy (Equation 1) between their profiles. Find the closest pair.

*Agglomeration:*

While (#classes >1) do:

1. Join the two closest classes into a new class, represented by a new node in the tree. Add edges from the new node to each daughter node.

2. Construct a profile for the new class based on the joint MSA.

3. Compute the distance between this new class and other classes (Equation 1).

4. Compute the encoding cost of this partition, under a Dirichlet mixture density (Equation 2).

*Output:*

1. Hierarchical tree.

2. Predicted subfamilies, corresponding to the stage in the agglomeration having the lowest encoding cost.

*Subtree profile construction:* each class (individual sequence or set of sequences) is represented by a profile [62] of amino acid distributions. Profiles are estimated using Dirichlet mixture densities [37], which helps generalize the amino acid distributions to include probabilities for similar amino acids at each position.

*Distances between profiles:* the distance function between profiles is a symmetrized form of relative entropy [38] summed over the alignment length (the *total relative entropy* or *TRE*). The TRE for profiles *p* and *q* is averaged over all columns *c*, such that both *p_c_* and *q_c_* distributions are based on columns with ≥1 amino acid (i.e., neither column contains only gap characters).

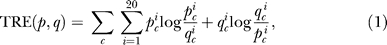
where *p_c_^i^* is the probability of amino acid *i* at position *c* in profile *p*.

*Encoding cost determination of subfamily decomposition:* subfamily identification is achieved using minimum-description–length principles to determine a cut of the tree into subtrees. There are a very large number of potential tree cuts; we employ a heuristic that examines only those partitions produced during the agglomerative clustering. At each iteration in the tree-building process, we have a forest of distinct subtrees that correspond to a particular cut of the tree. For each of these cuts, we evaluate the cost to encode the current set of subtree alignments under a Dirichlet mixture density [36,37]. The encoding cost function, assuming all subfamilies are independent, is defined as

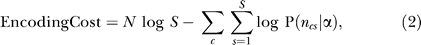
where *N* is the number of sequences in the MSA, *S* is the current number of subtrees, and P(*n_cs_* | *α*) is the probability of *n_cs_*, the ordered vector of observed amino acids for subfamily *s* at column *c*, under the Dirichlet mixture density *α*.

P(*n_cs_* | *α*) is obtained by integrating out the multinomial parameter *θ* from the model (see [63] for a review of the relevant mathematics). The canonical formulation for this quantity is

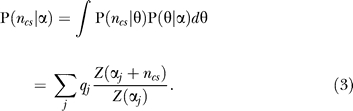
In this equation, *Z* is the normalizing constant for the Dirichlet distribution, *α_j_* is the *j* ^th^ component of *α*, and *q_j_* is its mixture coefficient.

The encoding cost function has two components: the first term is the cost to encode the subfamily labels for each sequence; the second term is the cost to encode each of the subtree alignments for that stage in the agglomeration. The two terms have opposite effects. The first term is large at program commencement when the number of subfamilies is largest, and reduces at each iteration, until it reaches zero at program termination, when there is one subfamily. The second term is minimized when the sequences within each subfamily are very similar to each other. At program commencement, for an input MSA with *N* sequences, there will be *N* separate subfamily alignments to be encoded. As the algorithm continues, the number of subfamilies decreases, until at program termination there is a single subfamily. As very similar sequences are joined into subtrees, the encoding cost decreases. For most protein families, the encoding cost curve decreases steadily to a minimum and then increases as subtrees with different amino acid preferences are joined. The stage in the agglomeration for which the encoding cost is minimal is used to determine a cut of the tree into subfamilies. See Figure 4.

### Sequence weighting in subfamily profile construction.

Sequence weighting is a standard approach in profile and HMM construction to prevent large subgroups from dominating amino acid distributions [64]. We use sequence weighting in both SCI-PHY tree construction and in SHMM construction.

We estimate sequence weights for each subfamily in a two-step process. In the first step, we estimate the total number of independent counts (NIC) in the alignment, as follows. We compute for every position in the alignment the frequency of the most frequent amino acid (ignoring gaps) to derive the positional conservation propensity. We then find the average of this value over all columns having at least one amino acid to obtain the overall conservation propensity (*P_cons_*). The NIC for the alignment can then be defined as 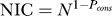 , where *N* is the number of sequences in the alignment. This has the effect of producing an NIC of 1 when the sequences in the alignment are 100% identical, and having the NIC approach *N* as the diversity in the alignment increases. In the second step, the relative weights for sequences in each subfamily can then be derived independently (e.g., [64]), normalizing them to sum to the NIC for that subfamily.

### Subfamily HMM construction.

The input to subfamily HMM construction is an MSA and a decomposition of the alignment into subfamilies. We construct SHMMs in a multi-step process.

*Subfamily HMM architecture and transition parameters:* first, we construct a family HMM using the entire input MSA as input and the SAM w0.5 software, developed by the University of California Santa Cruz (UCSC) Computational Biology group to optimize hidden Markov models for remote homolog detection [56]. To construct the subfamily HMM, the overall architecture and transition parameters in the family HMM are copied without modification for each SHMM. Keeping the overall architecture fixed enables sequence alignments to any SHMM to be easily mapped to the family as a whole.

*Match-state amino acid emission parameter estimation:* we first identify all positions that are conserved across the family as a whole, allowing gaps. The probability distribution for these positions is taken from the family HMM and fixed within all subfamilies, representing their common functional or structural role. This heuristic also enables small subfamilies to have conserved distributions at positions that clearly define the family as a whole. Next, we identify columns in subfamily alignments containing only gap characters; the match state distributions for these positions are copied from the corresponding match state of the general HMM. This heuristic compensates for any fragmentary or partially aligning sequences included in the MSA. For all other columns, we estimate the amino acid distributions for each subfamily *s* using an information-sharing protocol enabled by the use of Dirichlet mixture densities. For notational simplicity, we suppress the *c* index in the equations that follow.

*Step 1*. We estimate a posterior Dirichlet mixture density 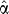 from the prior density *α* using the weighted amino acid counts in *s*. The mixture coefficients *q* are updated by setting each component *j* to its posterior probability given the observed (weighted) counts *n_s_*:

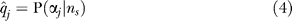



The component parameters 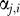 are also updated to include the *weighted counts* 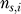 of observed amino acids:

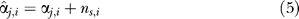
where *i* ranges over the twenty amino acids.

*Step 2*. We then include counts from other subfamilies *s′* in proportion to their probability under 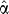 , obtaining the vector of total training counts *t* as

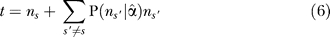



*Step 3*. Finally, we obtain a posterior estimate of the amino acid distribution from the original *α* using *t* rather than *n*

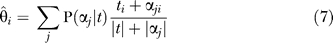
where |*x*| indicates the magnitude of *x*.

Thus, the generalization capability of subfamily HMMs is enhanced by adding in weighted counts from subfamilies having similar amino acids at corresponding positions.

### Details of experimental validation.

*Construction of benchmark datasets.* SCOP-PFAM515: protein families from the PFAM [40] resource were selected according to the following criteria: (1) the PFAM alignment had to match exactly one SCOP superfamily, and (2) SCI-PHY analysis of the alignment had to detect ≥2 subfamilies. The first criterion was determined based on scoring the Astral PDB90 dataset of structural domains [65] against all the HMMs in PFAM using the HMMER software (version 2.3.2) [66], and accepting only those PFAM families matching exactly one SCOP superfamily within the family gathering threshold. The second criterion was chosen to ensure that comparing subfamily and family HMM performance would be informative. Since each SCOP fold can contain numerous superfamilies, PFAM families meeting these criteria were filtered based on the top HMM score to select a single representative of each SCOP fold, to ensure that no fold dominated the results. This produced a set of 515 PFAM families. PFAM full alignments for these families were edited to remove sequences with >95% identity to other sequences in the MSA, columns with >70% gaps, and fragmentary sequences having >30% gap characters in the remaining columns.

*EC dataset construction*: the SCOP-PFAM515 families were examined to find those containing multiple enzymatic functions based on EC numbers obtained from the UniprotKB [67] database for sequences aligning over >75% of their lengths. The requirement of a minimum fractional alignment was included to prevent an assigned enzymatic function being associated with a domain not represented by the PFAM family selected. 57 families having multiple EC numbers remained after this procedure.

Since the majority of the sequences in each of these families had no assigned EC numbers, subfamily clustering methods were performed on the full (edited) alignments, but accuracy was assessed using only annotated sequences aligning over >75% of their lengths.

EXPERT: we selected the enolase and crotonase enzyme families from the SFLD [48], the amine and secretin families from the GPCRDB [49], and the NHR family from NucleaRDB [50]. For the enolase and crotonase families, the full-length sequences were aligned to the structural alignment for the families downloaded from the SFLD Web site. The common domain in the aminergic GPCRs was identified by aligning the full-length sequences from GPCRDB to the PFAM 7tm_1 HMM (PF00001). Similarly, the PFAM 7tm_2 HMM (PF00002) was used for the secretin-like GPCRs. The ligand-binding domain of nuclear hormone receptors was identified using the PFAM Hormone_recep HMM (PF00104). Next, we constructed an MSA for the identified domains from each family using the MUSCLE software [68]. Finally, we masked columns with ≥70% gap characters and made the alignment non-redundant at 98% identity (by restricting the alignment to a representative set such that no two sequences had ≥98% identity).

*Evaluating predicted subfamilies relative to expert-defined subtypes.* Several scoring functions were developed to enable us to evaluate the performance of predictive methods. Subfamily purity is measured as the fraction of subfamilies that contain only one expert subtype or EC number. The VI is a distance metric on partitions [53]. As such, it obeys the triangle inequality: *VI*(*A*,*B*) + *VI*(*B*,*C*) ≥ *VI*(*A*,*C*) for partitions *A*,*B*,*C*. Given two partitions, the VI index measures the amount of information in each partition that is not shared between them. It is calculated as

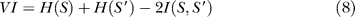
where *H* is the entropy of a partition, and *I* is the mutual information between two partitions:

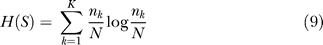


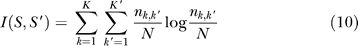



Here, *n_k_* is the number of items in cluster *k* of partition *S*, *n_k,k′_* is the number of overlapping items between cluster *k* in partition *S* and cluster *k′* in partition *S′, K* and *K′* are the total number of clusters in partitions *S* and *S′*, respectively, and *N* is the total number of items in the set.

The *Edit Distance* is defined as the minimum number of split or merge operations required to transform one partition into the other. A split or merge affecting multiple data points is considered one operation. For instance, two clusters containing five sequences may be merged into one ten-sequence cluster with an edit distance of one. The edit distance between a reference and a predicted partition with clusters *k* and *k′*, respectively, is calculated as

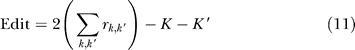
where *r_k,k′_* equals 1 if clusters *k* and *k′* have items in common, and zero otherwise, and *K* and *K′* are the number of clusters in each partition. Like the VI distance, the edit distance is a metric on partitions. Identical partitions will have an edit distance of zero. The edit distance is bounded by 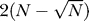 .

*SHMM construction and performance:* HMM construction and scoring: we used the UCSC Sequence Alignment and Modeling (SAM) software system [69,70] to score sequences in both fold prediction and classification experiments. All family HMMs were estimated using the SAM w0.5 software. Reverse scores for SHMMs and family HMMs were derived using the hmmscore program and local–local scoring (SAM parameter -sw 2). Family HMM e-values for sequences were calculated using the SAM recommended protocol: family HMMs were calibrated with the hmmscore program using the -calibrate option prior to scoring. Raw SHMM scores were obtained by scoring a sequence against all SHMMs in the family and retaining the top score. SHMM e-values were derived by fitting EVD parameters to raw SHMM scores for randomly generated sequences, using a maximum likelihood approach similar to that implemented in HMMER [71,72]. E-values were calculated based on a fixed database size of 10^5^. Weighted coverage and ROC curves were calculated by normalizing the contribution of each true positive by the size of its superfamily, so that all superfamilies contributed equally to the dataset [73].

*Subfamily classification and novel subtype detection:* for leave-one-out experiments, we chose ten sequences at random from each family, which were removed and tested separately. Sequences drawn from singleton subfamilies were replaced and not used. The modified alignment was then used to construct a new set of SHMMs, keeping the original SCI-PHY subfamily decomposition. To simulate classification of remotely related sequences, all sequences having identity greater than a specific cutoff to the withheld sequence were also removed from the alignment. We tested cutoffs of 30%, 40%, 50%, 60%, and 70% identity (see Table 4). In some cases, there were no sequences below the threshold within the subfamily; these were removed from the test. For the BLAST method, test sequences were assigned to the subfamily of the highest-scoring hit. To enable direct comparison, the Hannenhalli and Russell sub-profile method was re-implemented to use HMMs constructed from SAM w0.5 software rather than HMMER as in [43].

In the novel subtype detection experiments, we removed up to five complete subfamilies at random from each family, ignoring families with only two subfamilies (preventing the case where regression curves would have been trained with no negative examples). Results were normalized by subfamily size. Logistic regression parameters were fit using the iteratively re-weighted least squares (IRLS, [74]) algorithm (implemented in R [75]).

## Supporting Information

Dataset S1 De Novo Subfamily Identification on the EXPERT and EC Datasets (69 KB PDF)

Dataset S2 Assessing SCI-PHY Agreement with Phylogenetic Clades (136 KB PDF)

Dataset S3 Adrenoreceptor GPCRs (106 KB PDF)

Dataset S4 Comparison of “Informed” versus “Naïve” Subfamily HMMs (37 KB PDF)

Dataset S5 Novel Sequence Classification Using the EXPERT Dataset (33 KB PDF)

Dataset S6 Annotation Transfer and Detecting Annotation Error (47 KB PDF)

## Abbreviations

EC: enzyme commission
EPQ: errors per query
EVD: extreme value distribution
GO: gene ontology
GPCRDB: G-protein coupled receptor database
HMM: hidden Markov model
ML: maximum likelihood
MR: majority rule
MSA: multiple sequence alignment
NHR: nuclear hormone receptor
NIC: number of independent counts
NJ: neighbor joining
SCI-PHY: Subfamily Classification in Phylogenomics
SCOP: Structural Classification of Proteins
SFLD: Structure Function Linkage Database
SHMM: subfamily hidden Markov model
SVM: support vector machine
TRE: total relative entropy
VI: variation of information
