# Supplementary material for: Automated Protein Subfamily Identification and Classification
Source: PLoS Comput Biol. 2007 Aug 17;3(8):e160. doi: 10.1371/journal.pcbi.0030160 (PMC1950344; doi:10.1371/journal.pcbi.0030160)
Supplement: Supplementary file 1 [file pcbi.0030160.sd001.pdf]

S1 *De novo* subfamily identification on the EXPERT and EC datasets

We present the results for additional CD-HIT parameterizations not given in the main text (Table S1. The results continue the trends identified in the paper. CD-HIT 50 generally gives similar purity to SCI-PHY, but worse distance scores in every case except Enolase (for the edit distance) and NHR L3 (where it achieves the same VI distance). From these results, it appears that CD-HIT 60 provides a better overall breakdown than CD-HIT 70, since its purity scores are only slightly worse (and, interestingly, actually better for NHR Level 3), while its distance scores are significantly better. This reflects the diminishing returns in purity afforded by the higher percent identity parameterizations of CD-HIT. The NCUT algorithm (Abascal and Valencia, 2002) performed very inconsistently, and while its purity score of 0.875 for the aminergic GPCR family appears to be high, the one impur subfamily contained 89% of sequences in the family, including members of every level 1 subtype.

The expanded results include all CD-HIT parameterizations (Table S2), and are highly similar to the reported results in the text. The purity scores show diminishing returns as the identity threshold is raised, while the distance scores degrade significantly with each new level.

	Amine L1	Amine L2	Crotonase	Enolase	NHR L1	NHR L2	NHR L3	Secretin
Subtypes/Seqs	7/358	31/358	10/365	8/472	8/412	27/412	77/412	15/153
Pure/Total SF								
SCI-PHY	36/37	32/37	15/16	26/26	29/29	28/29	11/29	14/16
SECATOR	10/14	6/14	3/7	8/9	3/5	1/5	0/5	3/6
NCUT	7/8	7/8	14/16	65/66	24/25	22/25	10/25	8/10
CD-HIT 40	22/33	16/33	47/47	16/18	28/28	24/28	9/28	7/11
CD-HIT 50	50/50	45/50	66/66	34/34	35/36	35/36	17/36	15/17
CD-HIT 60	60/60	54/60	70/70	52/52	47/47	47/47	46/47	25/28
CD-HIT 70	67/67	66/67	65/65	73/73	57/57	57/57	41/57	31/31
CD-HIT 80	72/72	72/72	50/50	78/78	68/68	68/68	61/68	36/36
CD-HIT 90	68/68	68/68	33/33	69/69	83/83	83/83	79/83	34/34
Edit Distance								
SCI-PHY	38	36	32	70	38	21	54	15
SECATOR	49	61	53	32	6	23	71	9
NCUT	40	62	23	99	30	23	57	14
CD-HIT 40	64	84	55	28	30	21	69	25
CD-HIT 50	55	51	112	52	44	25	64	18
CD-HIT 60	74	64	160	99	64	45	68	30
CD-HIT 70	107	85	213	177	90	71	70	35
CD-HIT 80	142	118	264	252	133	114	89	50
CD-HIT 90	205	181	312	343	212	193	154	82
VI Distance								
SCI-PHY	1.55	0.90	1.05	1.37	1.62	0.39	0.95	0.56
SECATOR	1.44	1.14	1.29	0.87	0.43	1.32	2.39	0.91
NCUT	2.08	3.01	0.63	2.75	1.38	0.57	1.21	1.15
CD-HIT 40	1.94	1.37	2.28	0.56	1.47	0.45	1.16	1.25
CD-HIT 50	1.85	0.96	2.91	1.44	1.80	0.50	0.95	0.79
CD-HIT 60	2.14	1.09	3.37	2.40	2.16	0.86	0.80	0.89
CD-HIT 70	2.53	1.31	3.77	3.19	2.55	1.25	0.70	0.99
CD-HIT 80	2.86	1.62	4.06	3.77	2.96	1.66	0.86	1.33
CD-HIT 90	3.32	2.07	4.32	4.33	3.58	2.28	1.27	1.84
Singletons								
SCI-PHY	6	6	22	52	17	17	17	6
SECATOR	34	34	46	29	5	5	5	0
NCUT	27	27	9	35	9	9	9	3
CD-HIT 40	4	4	18	14	10	10	10	3
CD-HIT 50	12	12	56	26	14	14	14	6
CD-HIT 60	21	21	100	55	25	25	25	9
CD-HIT 70	47	47	158	112	41	41	41	19
CD-HIT 80	77	77	224	182	73	73	73	29
CD-HIT 90	144	144	289	282	137	137	137	63

Table S1: ***De novo* subfamily identification for the EXPERT set.** We compared the performance of SCI-PHY, SECATOR and the six CD-HIT parameterizations on the eight functional classifications. Subtypes/Sequences: the number of expert-derived subtypes / the number of sequences in each classification. Pure/Total SF: the fraction of pure non-singleton subfamilies / the total number of non-singleton subfamilies for each method. Singletons: the number of single-sequence clusters for each method.

	SECATOR	SCI-PHY	CDHIT 40	CDHIT 50	CDHIT 60	CDHIT 70	CDHIT 80	CDHIT 90
SECATOR	P: 0.71<br>V: 0.91<br>E: 8.9	(0.003)<br>0.018<br>0.005	(1.9×10^{-5})<br>4.0×10^{-7}<br>6.3×10^{-7}	(1.8×10^{-7})<br>1.5×10^{-9}<br>4.7×10^{-8}	(2.6×10^{-7})<br>1.0×10^{-9}<br>5.0×10^{-9}	(1.9×10^{-7})<br>5.7×10^{-10}<br>6.4×10^{-10}	(1.9×10^{-7})<br>4.1×10^{-10}<br>4.4×10^{-10}	(4.4×10^{-7})<br>3.6×10^{-10}<br>5.3×10^{-10}
SCI-PHY		P: 0.80<br>V: 1.07<br>E: 10.8	(0.007)<br>5.0×10^{-8}<br>3.2×10^{-6}	(3.3×10^{-6})<br>2.9×10^{-8}<br>1.9×10^{-9}	(3.3×10^{-6})<br>4.4×10^{-9}<br>6.9×10^{-10}	(1.2×10^{-6})<br>2.3×10^{-9}<br>6.4×10^{-10}	(1.2×10^{-6})<br>9.1×10^{-10}<br>6.2×10^{-10}	(1.2×10^{-6})<br>7.7×10^{-10}<br>6.0×10^{-10}
CDHIT 40			P: 0.88<br>V: 1.44<br>E: 14.7	(4.8×10^{-5})<br>1.8×10^{-4}<br>5.3×10^{-7}	(2.0×10^{-5})<br>2.6×10^{-7}<br>1.4×10^{-8}	(2.7×10^{-6})<br>3.8×10^{-8}<br>7.3×10^{-9}	(3.7×10^{-6})<br>7.5×10^{-9}<br>5.1×10^{-9}	(1.4×10^{-5})<br>4.4×10^{-9}<br>3.4×10^{-9}
CDHIT 50				P: 0.94<br>V: 1.66<br>E: 19.4	(0.002)<br>5.8×10^{-8}<br>2.5×10^{-8}	(6.6×10^{-4})<br>9.1×10^{-9}<br>8.8×10^{-9}	(6.6×10^{-4})<br>2.8×10^{-9}<br>2.9×10^{-9}	(0.001)<br>2.5×10^{-9}<br>2.5×10^{-9}
CDHIT 60					P: 0.96<br>V: 1.89<br>E: 24.5	(0.01)<br>1.1×10^{-7}<br>2.0×10^{-8}	(0.002)<br>5.1×10^{-9}<br>4.5×10^{-9}	(0.008)<br>2.5×10^{-9}<br>2.5×10^{-9}
CDHIT 70						P: 0.97<br>V: 2.07<br>E: 30.0	(0.03)<br>6.5×10^{-8}<br>5.4×10^{-8}	(0.06)<br>2.7×10^{-9}<br>5.2×10^{-9}
CDHIT 80							P: 0.98<br>V: 2.23<br>E: 35.3	0.59<br>1.8×10^{-7}<br>1.8×10^{-7}
CDHIT 90								P: 0.99<br>V: 2.35<br>E: 40.4

Table S2: Wilcoxon signed rank tests for *de novo* subfamily detection on the EC dataset. Diagonal entries are the mean scores for the method. P: Purity score; 1.0 is perfect, indicating that all subfamilies contain sequences from a single EC class. V: Variation of information distance; E: edit distance. A distance of zero in either case indicates that predicted subfamilies are identical to EC classes. Upper-diagonal entries are the Wilcoxon signed rank p-values between methods for the corresponding score. In comparison of two methods for a particular score, parentheses around the p-value indicates that the method listed in the column is better; plain values indicate that the row method is better.
