# Supplementary material for: Automated Protein Subfamily Identification and Classification
Source: PLoS Comput Biol. 2007 Aug 17;3(8):e160. doi: 10.1371/journal.pcbi.0030160 (PMC1950344; doi:10.1371/journal.pcbi.0030160)
Supplement: Supplementary file 2 [file pcbi.0030160.sd002.pdf]

S2 Assessing SCI-PHY agreement with phylogenetic clades

We used a subset of 170 families taken from SCOP-PFAM515 to assess SCI-PHY subfamily correspondence with conserved clades found in phylogenetic trees. Due to the computational complexity of phylogenetic tree construction, the 515 set was filtered to remove large families (i.e., with >200 sequences after removal of fragmentary and highly similar sequences). We also removed very small families with <50 sequences, as these often had very few subfamilies, making comparisons almost trivial. For each multiple sequence alignment, we constructed four standard phylogenetic trees: neighbor-joining (NJ), parsimony, maximum likelihood (ML), and a bootstrapped NJ tree (NJ500). Two additional consensus trees were estimated: one using a majority rule consensus of the parsimony, ML and NJ trees (the MR tree), and another consensus tree of the parsimony, ML and bootstrapped NJ500 trees (MR500).

We developed two measures of similarity between the SCI-PHY subfamily decomposition and phylogenetic trees. An obvious measure of correspondence between a subfamily and a rooted phylogenetic tree is whether the sequences in a subfamily form a monophyletic subtree within the tree; we termed this measure *support*. Alternatively, a subfamily can be considered *consistent* with a tree if the tree topology can be resolved in such a way as to render support to the subfamily (see Figure S1). Note that these measures are identical when the tree is fully resolved (bifurcating). Consistency and support are complementary measures of the compatibility of the subfamily with the tree topology. Support is a natural way to compare a subfamily to a tree, but it is a stringent measure; if the tree topology is ambiguous, a subfamily may not have support even though it may in fact be the correct evolutionary group. It is reasonable to differentiate such situations from those in which the subfamily is clearly in conflict with the tree. Our consistency measure allows such ambiguity to be resolved in favor of the subfamily.

We assessed each non-singleton SCI-PHY subfamily with respect to the various trees estimated (Figure S2). Our results suggest that SCI-PHY subfamilies are reliable evolutionary classifications. SCI-PHY subfamilies showed clear agreement with all phylogenetic methods, receiving support from each tree topology and their consensii 70-75% of the time. SCI-PHY subfamilies received the highest consistency and the lowest support scores from consensus trees, due to ambiguity regarding the true tree topology. However, support scores did not differ greatly between any of the tree methods or their consensii, and results from the NJ500 and MR500 trees showed that most subfamilies were very well supported by the alignment data: 70% of SCI-PHY subfamilies were supported by the NJ500 trees, as opposed to 76% for the non-bootstrapped NJ trees. Measures of consistency were higher: 89% of SCI-PHY subfamilies were consistent with the bootstrapped NJ500 trees, compared to 76% before bootstrapping. This shows that most of the conflicting SCI-PHY subfamilies were also in areas of low bootstrap support, and may in fact be quite reasonable biologically.

We relaxed our measures to ignore cases in which the SCI-PHY decomposition differed from the tree topology only in the placement of singleton subfamilies (see Figure S3, Type II). In these cases, the SCI-PHY classification was reasonable; it did not bring together phylogenetically distant subtrees, but merely excluded individual sequences incorrectly (assuming the phylogenetic tree used as a reference represents a "true" evolutionary classification). Scores improved considerably: 92% of SCI-PHY subfamilies were consistent with the NJ500 trees when singletons were ignored (Figure S2, *Support without Singletons* and *Consistency without Singletons*). These experiments

indicate significant support for SCI-PHY subfamilies by standard methods of evolutionary tree construction.

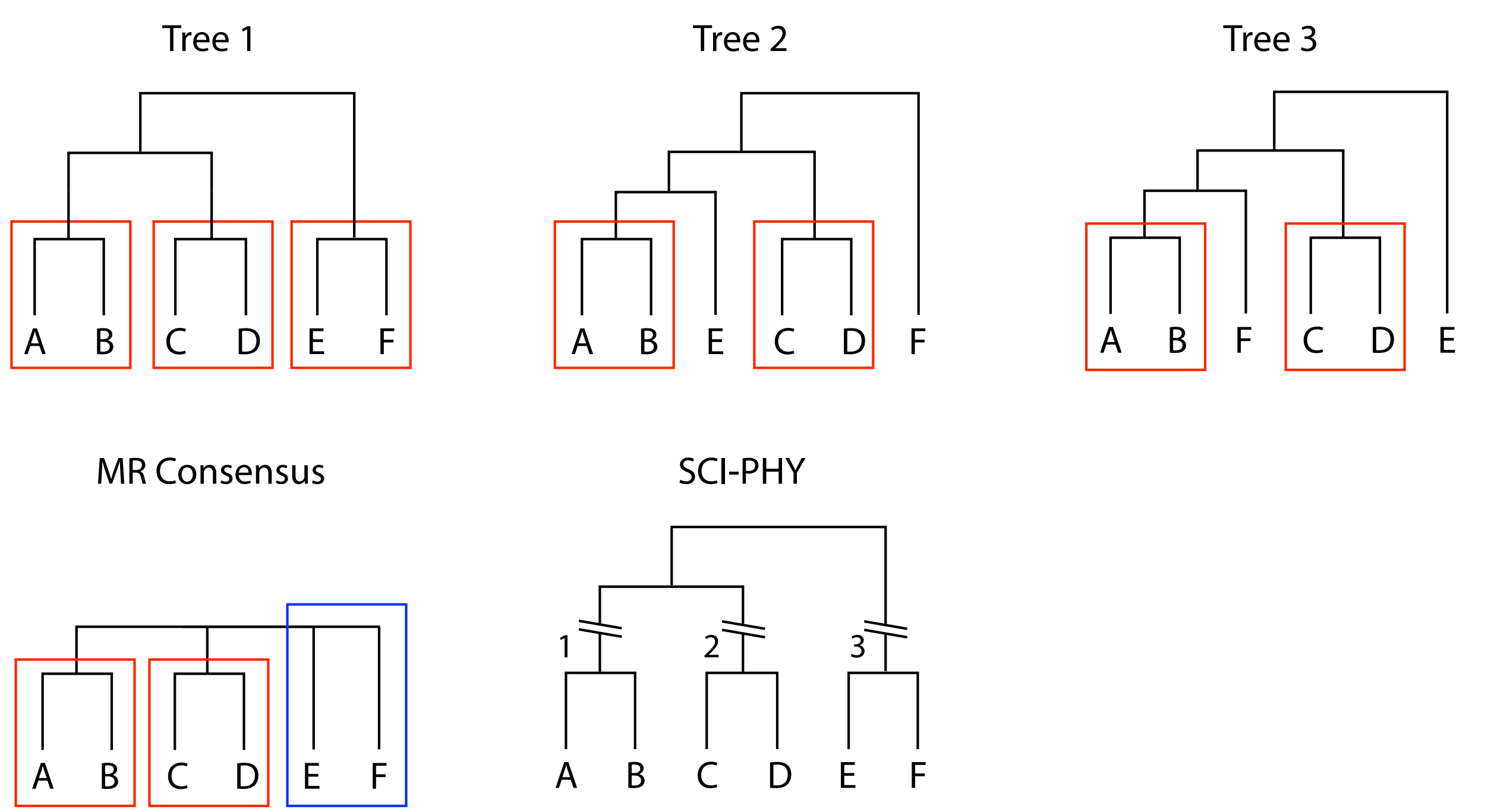

Figure S1: **Subfamily support and consistency.** SCI-PHY subfamilies are marked with broken lines. Subfamilies 1 and 2 have support from all three trees and the consensus tree, while subfamily 3 has support only from tree 1 (red boxes). However, subfamily 3 is consistent with the consensus tree, since the topology does not explicitly separate sequences E and F (blue box).

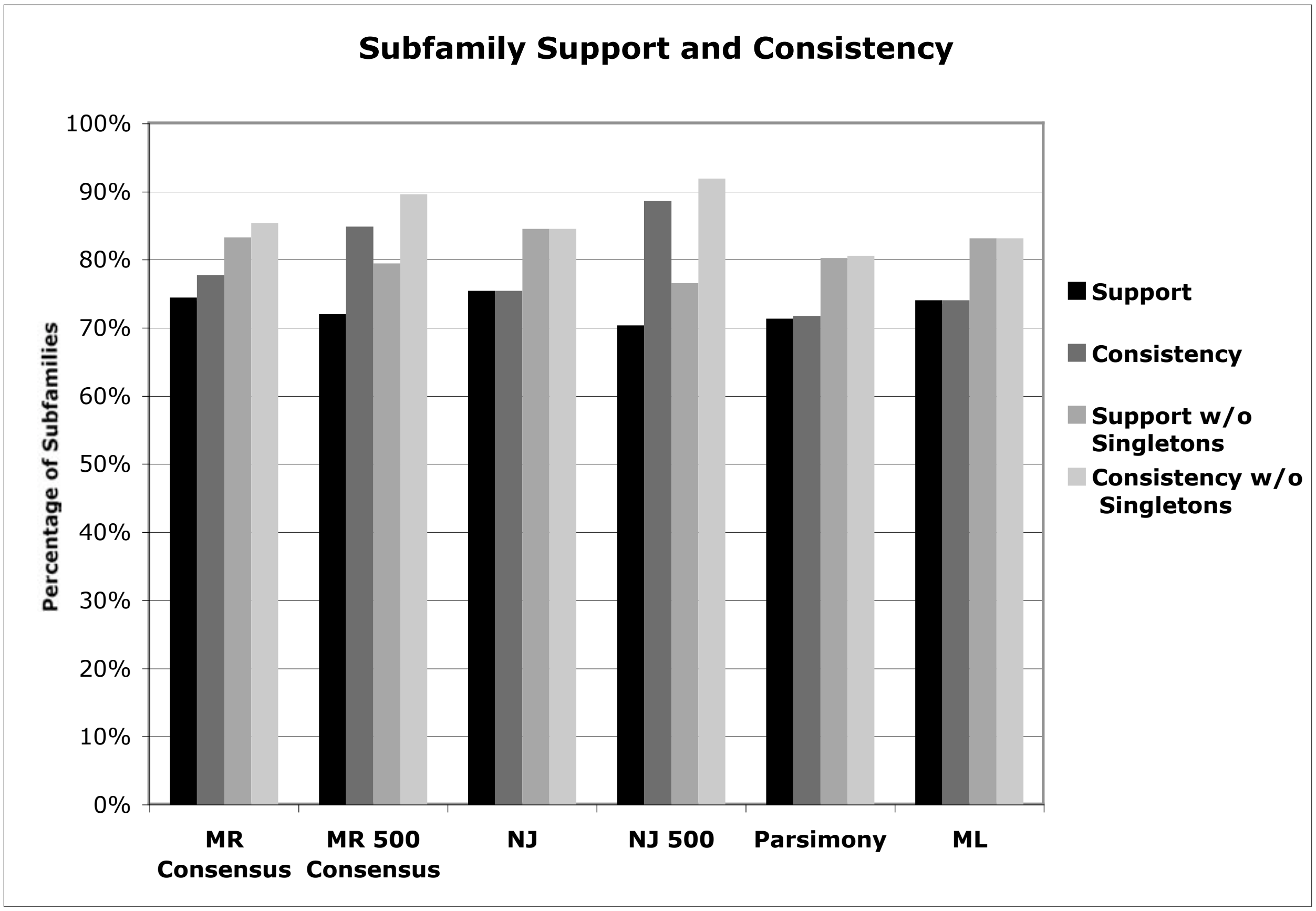

Figure S2: **Comparison of SCI-PHY subfamilies with phylogenetic trees from 170 families.** SCI-PHY subfamilies are given high support from all phylogenetic methods. SCI-PHY also obtains high consistency with consensus trees, reflective of the ambiguity regarding tree topology. "Support (Consistency) without singletons" was calculated by ignoring errors due to the placement of singletons (Figure S3).

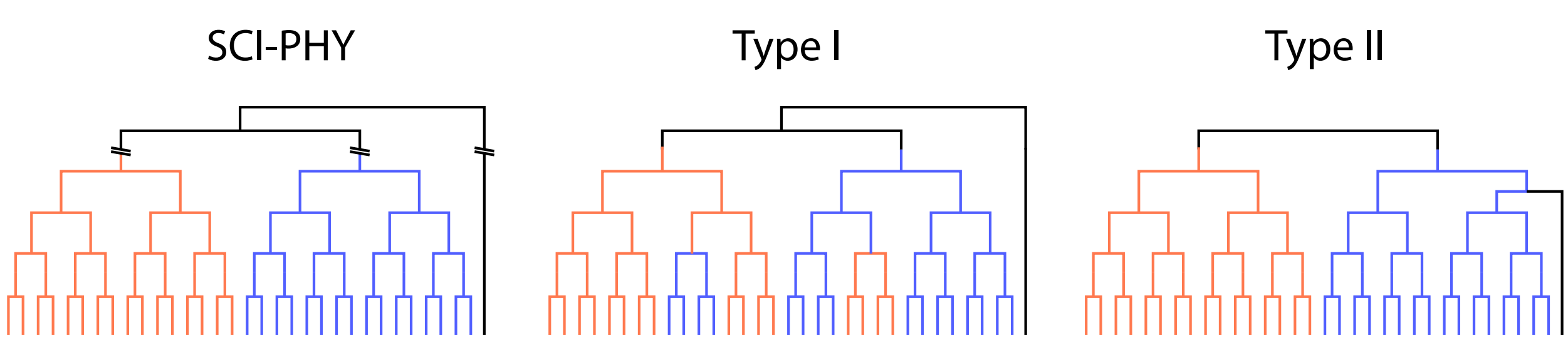

Figure S3: **Types of subfamily errors.** In type I, SCI-PHY has joined sequences from two different parts of the tree together into one subfamily. In type II, however, the subtrees match the SCI-PHY classification perfectly, except that SCI-PHY has incorrectly classified one of the sequences into its own subfamily. We relaxed the support and consistency measures to ignore the latter types of error.
