# Supplementary material for: Automated Protein Subfamily Identification and Classification
Source: PLoS Comput Biol. 2007 Aug 17;3(8):e160. doi: 10.1371/journal.pcbi.0030160 (PMC1950344; doi:10.1371/journal.pcbi.0030160)
Supplement: Supplementary file 3 [file pcbi.0030160.sd003.pdf]

S3 Adreno-receptor GPCRs

A detailed analysis of the performance of the different methods on the adreno-receptor subtype of the aminergic GPCR family clearly illustrates the challenges facing *de novo* subfamily identification (Figure S4). Aminergic receptors are seven-transmembrane receptors that recognize a diverse set of biogenic amine ligands (e.g., dopamine, histamine and serotonin). This class of GPCRs is of great biomedical interest as it contains a significant fraction of pharmaceutical targets. The GPCRDB provided two levels of classification for the aminergic receptors; level 1 contained seven expert subtypes, which were further subdivided at level 2 into 31 subtypes. Results for the level 1 adreno-receptor subtype illustrate the performance characteristics of the different algorithms.

The adreno-receptors were classified into six level 2 subtypes: Alpha1, Alpha2, Beta1, Beta2, Beta3 and Beta4. SCI-PHY divided the adreno-receptors into three subfamilies, achieving perfect purity at the level 1 classification. Two of the three subfamilies are also pure at level 2 (the Alpha1 and Alpha2 subtypes), but the third combined all four Beta subtypes together (although they were separated into sibling subtrees within the SCI-PHY subfamily).

Secator divided the adreno-receptors in a similar manner, although only one of the three subfamilies was pure; octopamine sequences were joined with the Alpha1 subfamily, and dopamine receptors into the Beta subfamily. This is presumably due to an overly coarse cut of the tree, and this pattern is repeated in the other EXPERT families: Secator subfamilies often represent a merging of several SCI-PHY subfamilies, causing low purity.

The Ncut subdivision is not shown; the method placed 61 of the 72 aminergic receptor sequences into one subfamily (which contained sequences from every level 1 subtype; 317 sequences total), and also created seven singletons and two subfamilies containing two sequences each.

CD-HIT40 divided the adreno-receptors into nine subfamilies, seven of which contained sequences from outside the adreno-receptor class, and was the worst performer in this case, having a higher VI distance than even CD-HIT70. CD-HIT70 was the only method other than SCI-PHY to perfectly separate the adreno-receptors from the rest of the family. However, it greatly over-divided them, creating 18 subfamilies, five of which were singletons.

This example illustrates the problems inherent in methods that use a simple percent identity cutoff to assess functional similarity. The performance of CD-HIT40 shows that the adreno-receptors do not fall into clearly defined identity groups, and highlights SCI-PHY's ability to partition sequences based on differing conservation within groups.

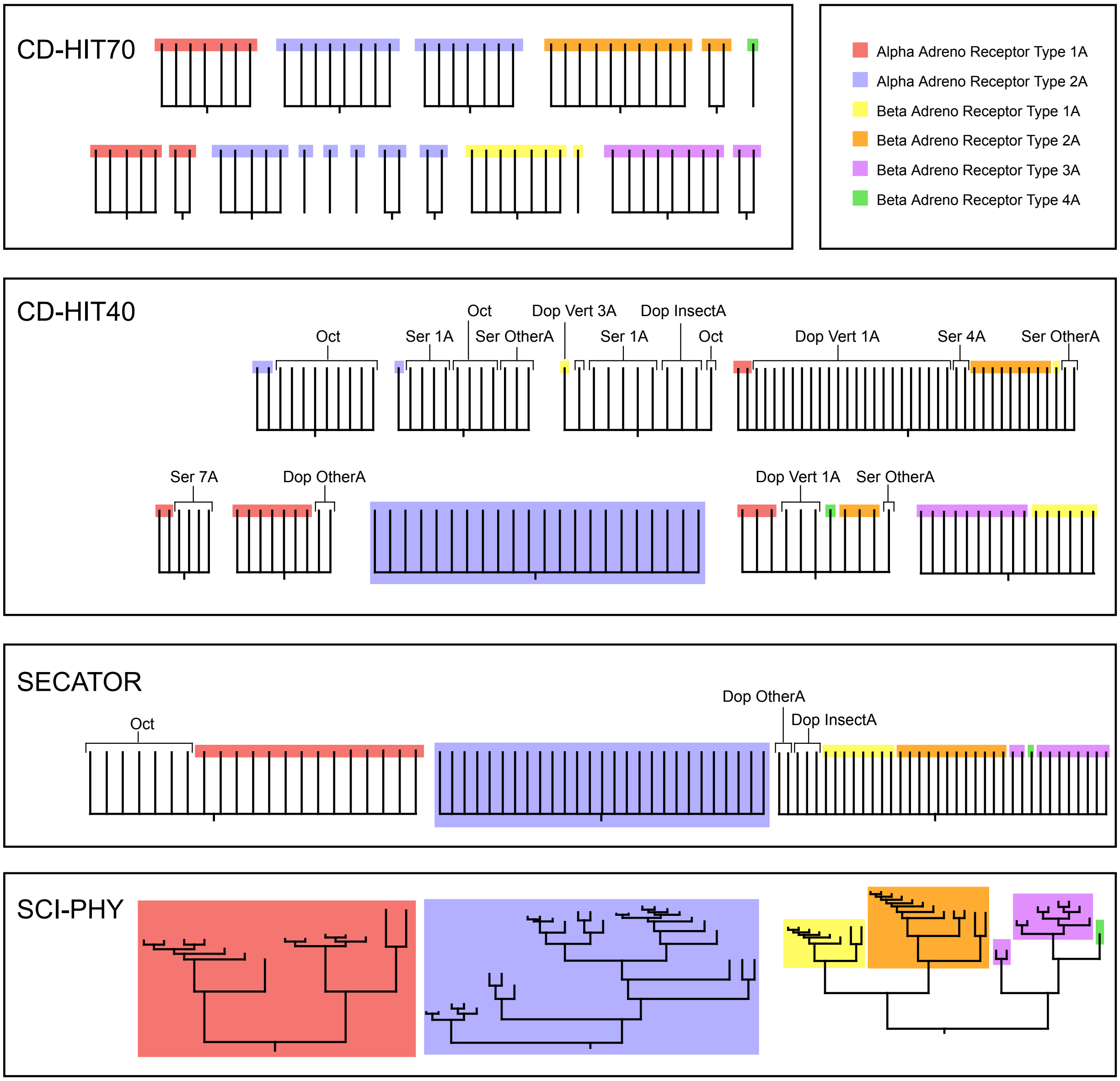

Figure S4: **Subfamily Detection in the Amine GPCRs.** We display results for the GPCRDB level 1 adreno-receptor subtype, which is split into 6 subtypes in level 2. Each of the six subtypes is marked by colored boxes. Sequences from other subtypes are labeled by name. *Dop*: Dopamine; *Dop Vert*: Dopamine Vertebrate; *Oct*: Octopamine; *Ser*: Serotonin. SCI-PHY and CD-HIT70 were the only methods to separate all the adreno-receptors from other subtypes, but neither was perfectly optimal with respect to the level 2 classification. SCI-PHY placed all the Beta subtypes together in one subfamily, while CD-HIT70's partition was too specific; it divided the group into 18 subfamilies.
