# Supplementary material for: Automated Protein Subfamily Identification and Classification
Source: PLoS Comput Biol. 2007 Aug 17;3(8):e160. doi: 10.1371/journal.pcbi.0030160 (PMC1950344; doi:10.1371/journal.pcbi.0030160)
Supplement: Supplementary file 4 [file pcbi.0030160.sd004.pdf]

S4 Comparison of 'informed' vs 'naive' subfamily HMMs

As shown in Table S3, homolog detection using subfamily HMMs constructed with our information sharing protocol ('informed' SHMMs) is much more sensitive on average than for SHMMs built using standard HMM training methods ('naive' SHMMs). The scores for informed SHMMs are uniformly better, achieving improved significance of nearly 9 orders of magnitude on average. This is due to the information-sharing protocol that adds counts from similar subfamilies during HMM training. The effect is more pronounced for small subfamilies; this reflects in the increased effect of information-sharing when observed amino acid counts are low, and allows excellent generalization to unseen family members, while maintaining subfamily specificity.

Subfamily Size	Naive	SHMM
All	R: -34.04<br>E: 1.7×10^{-10}	R: -53.57<br>E: 5.4×10^{-19}
< 10	R: -31.23<br>E: 2.7×10^{-9}	R: -53.74<br>E: 4.6×10^{-19}
< 5	R: -28.36<br>E: 4.8×10^{-8}	R: -52.01<br>E: 2.6×10^{-18}
1	R: -24.72<br>E: 1.8×10^{-6}	R: -50.65<br>E: 1.0×10^{-17}

Table S3: Average scores for 'naive' and 'informed' SHMMs. For each family in the SCOP515, true positive SCOP sequences for the family were scored against naive and informed SHMMs. The table lists the average family reverse scores (R) and E-values (E) for all subfamilies, subfamilies with less than 10 sequences, subfamilies with less than 5 sequences, and singletons.
