# Supplementary material for: Automated Protein Subfamily Identification and Classification
Source: PLoS Comput Biol. 2007 Aug 17;3(8):e160. doi: 10.1371/journal.pcbi.0030160 (PMC1950344; doi:10.1371/journal.pcbi.0030160)
Supplement: Supplementary file 5 [file pcbi.0030160.sd005.pdf]

## S5 Novel sequence classification using the EXPERT dataset

Novel sequence classification experiments were repeated using manually defined subfamilies from the EXPERT set (Table S4). All sequences in each family were tested, except for those sequences whose original subfamily was emptied due to removal of similar seqeunces. Overall results are quite similar to those for the larger SCOP-PFAM515 set, in that the BLAST, sub-profile and SHMM methods all performed comparably. Coarse classifications such as enolase, crotonase and NHR L1, in which subtypes were both well-populated and well separated, proved relatively easy for all methods at all levels of identity. The exception was the Amine family, in which accuracy dropped below 20% for the most difficult threshold of 30% identity. We believe this is due to the high diversity within the Amine L1 subtypes (on average, each subtype has a pair of sequences with just 20% identity), in combination with high similarity between subtypes. Interestingly, Amine L2 gave better results. This was most likely due to the small number of sequences tested. The secretin subtypes were not well-separated, and only 31 sequences were left to test at the 30% threshold. All three methods did well at this low threshold (the SHMM method performed perfectly), but at higher thresholds of 40% and 50%, performance decreased. This reflects the removal of 'difficult' sequences (those having quite similar homologs in other subtypes) at the lowest threshold. The same effect is seen in the NHR L3 classification.

| | Amine L1 | Amine L2 | Crotonase | Enolase | NHR L1 | NHR L2 | NHR L3 | Secretin |
|---|---|---|---|---|---|---|---|---|
| Avg Min Subtype %ID | 20.8 | 56.7 | 38.6 | 23.8 | 21.3 | 38.9 | 67.5 | 54.5 |
| Max Cross-subtype %ID | 49 | 72 | 41 | 47 | 41 | 49 | 97 | 64 |
| 30% | 297 | 73 | 271 | 265 | 397 | 174 | 42 | 31 |
| SHMM | 11.1 | 27.4 | 95.2 | 96.2 | 73.3 | 57.5 | 45.2 | 100.0 |
| Sub-profile | 16.2 | 34.2 | 98.5 | 98.1 | 73.6 | 19.0 | 23.8 | 83.9 |
| BLAST | 14.8 | 17.8 | 97.9 | 96.2 | 68.0 | 40.2 | 38.1 | 96.8 |
| 40% | 354 | 165 | 292 | 456 | 405 | 252 | 51 | 39 |
| SHMM | 33.3 | 47.9 | 99.0 | 95.8 | 94.1 | 60.3 | 45.1 | 66.7 |
| Sub-profile | 60.5 | 56.4 | 100.0 | 92.8 | 95.8 | 57.5 | 54.9 | 48.7 |
| BLAST | 62.4 | 66.1 | 99.7 | 93.0 | 93.8 | 69.0 | 45.1 | 56.4 |
| 50% | 358 | 198 | 344 | 472 | 408 | 256 | 68 | 78 |
| SHMM | 59.2 | 68.7 | 99.1 | 96.4 | 96.6 | 82.4 | 26.8 | 43.6 |
| Sub-profile | 74.3 | 69.1 | 100.0 | 94.1 | 96.3 | 83.6 | 36.8 | 44.9 |
| BLAST | 78.2 | 82.3 | 100.0 | 93.9 | 96.1 | 84.4 | 30.9 | 64.1 |
| 60% | 358 | 225 | 359 | 472 | 408 | 319 | 125 | 109 |
| SHMM | 79.3 | 77.8 | 99.2 | 97.5 | 100.0 | 94.4 | 52.8 | 73.4 |
| Sub-profile | 90.2 | 77.8 | 99.2 | 99.4 | 99.8 | 96.2 | 60.8 | 79.8 |
| BLAST | 88.5 | 81.3 | 100.0 | 98.7 | 99.5 | 97.2 | 72.0 | 76.1 |
| 70% | 358 | 300 | 361 | 472 | 408 | 379 | 244 | 117 |
| SHMM | 86.9 | 79.0 | 99.2 | 98.1 | 100.0 | 97.9 | 53.3 | 92.3 |
| Sub-profile | 92.2 | 84.3 | 99.2 | 99.8 | 99.8 | 98.7 | 60.7 | 93.2 |
| BLAST | 92.5 | 90.3 | 100.0 | 99.2 | 99.5 | 99.5 | 77.1 | 94.0 |

Table S4: Mean novel sequence classification accuracy on the EXPERT dataset, after removal of sequences having percent identity above the given threshold. Avg Min Subtype %ID is the average minimum percent identity within each subtype in the family. Max Cross-subtype %ID is the maximum percent identity between any two sequences in different subtypes. Numbers in the first row of each block indicate the number of sequences in each family tested at that threshold (in some cases, all sequences in the original subfamily were above the threshold and were removed; these cases were not tested).
