# Supplementary material for: Automated Protein Subfamily Identification and Classification
Source: PLoS Comput Biol. 2007 Aug 17;3(8):e160. doi: 10.1371/journal.pcbi.0030160 (PMC1950344; doi:10.1371/journal.pcbi.0030160)
Supplement: Supplementary file 6 [file pcbi.0030160.sd006.pdf]

S6 Annotation transfer and detecting annotation error

To illustrate biological discovery using our system of classification, we present several illustrations in which clustering sequences into SCI-PHY subfamilies enables functional assignment and detection of errors in function prediction.

By teasing apart subtypes within large protein families, our system enables both accurate function prediction and annotation error detection. For example, the amidohydrolase family of enzymes is a highly diverse group whose members exhibit a wide range of functions. SCI-PHY is able to separate these functions into discrete subfamilies, which can be used for functional annotation. Table S5 shows one subfamily from the SCI-PHY partition of the PhyloFacts book d1gkpa2 (available at http://phylogenomics.berkeley.edu/book/book_info.php?book=d1gkpa2). homologs of the hydantoinase SCOP superfamily. Within this subfamily, protein P20051 has been annotated as a dihydroorotase using experimental evidence. The subfamily clustering system allows us to transfer this function to the unannotated sequences in the subfamily, for highly precise and accurate function prediction.

When a protein family contains multiple functions, it is quite easy for individual sequences to be misannotated, or annotated with a very general function. We provide an example in Table S6, again from the amidohydrolase family. Six sequences (shown in red) within this subfamily of guanine deaminases have been annotated as chlorohydrolases or "cytosine/guanine deaminases." Based on the experimental evidence for sequences in the subfamily, these sequences are most likely misannotated. In addition, this example again highlights the effectiveness of SCI-PHY in facilitating annotation transfer: fifteen sequences without annotations in their description (and only high-level GO annotation) can be specifically labeled as guanine deaminases.

Accession	Source	Description	Species	GO function [evidence code]
caa30444	Genbank	unnamed protein product	Saccharomyces cerevisiae	
P20051	UniProt	Dihydroorotase (EC 3.5.2.3) (DHOase)	Saccharomyces cerevisiae	dihydroorotase activity [IMP] protein binding [IPI]
zp_00018287	Genbank	hypothetical protein	Chloroflexus aurantiacus	
Q9PIN6	UniProt	Dihydroorotase (EC 3.5.2.3)	Campylobacter jejuni	dihydroorotase activity [IEA] hydrolase activity [IEA]
eaa31733	Genbank	hypothetical protein	Neurospora crassa	
Q9UTI0	UniProt	Probable dihydroorotase (EC 3.5.2.3) (DHOase)	Schizosaccharomyces pombe	
aam46078	Genbank	dihydroorotase	Toxoplasma gondii	
P31301	UniProt	Dihydroorotase (EC 3.5.2.3) (DHOase)	Ustilago maydis	

Table S5: The N1852 subfamily from the SCI-PHY partition of sequences in the PhyloFacts book d1gkpa2, a member of the hydantoinase SCOP superfamily. The evidence code for each GO annotation are given in brackets after the term. P20051 (blue) has been annotated as a dihydroorotase using experimental evidence, which can be transferred to unannotated proteins within the subfamily (green).

Accession	Description	Species	GO function [evidence code]
Q831R9	Chlorohydrolase family protein	Enterococcus faecalis	hydrolase activity [IEA]
Q97MB6	Cytosine/guanine deaminase related protein	Clostridium acetobutylicum	hydrolase activity [IEA]
Q2WNG6	Cytosine/guanine deaminase related protein	Clostridium beijerincki NCIMB 8052	
Q5AFN9	Hypothetical protein	Candida albicans	
Q6C4L7	Similar to sp—Q07729 Saccharomyces cerevisiae YDL238c	Yarrowia lipolytica	hydrolase activity [IEA]
Q5AX44	Hypothetical protein	Emericella nidulans	
Q2U0Q0	Atrazine chlorohydrolase/guanine deaminase	Aspergillus oryzae	
Q4W9T7	Chlorohydrolase family protein	Aspergillus fumigatus	
Q4IBT5	Hypothetical protein	Gibberella zeae	
Q7SA53	Hypothetical protein NCU07309.1	Neurospora crassa	
Q6CQ62	Similar to sp—Q07729 Saccharomyces cerevisiae YDL238c singleton	Kluyveromyces lactis	hydrolase activity [IEA]
Q07729	Probable guanine deaminase (EC 3.5.4.3)	Saccharomyces cerevisiae	guanine deaminase activity [IDA] protein binding [IPI]
Q6FWH8	Similar to sp—Q07729 Saccharomyces cerevisiae YDL238c	Candida glabrata	hydrolase activity [IEA]
Q9VMY9	CG18143-PA (LD44207p) (RE08243p)	Drosophila melanogaster	protein binding [IPI]
Q7QI65	ENSANGP00000019574 (Fragment)	Anopheles gambiae str	
Q4PAC0	Hypothetical protein	Ustilago maydis	
Q2UHY8	Atrazine chlorohydrolase/guanine deaminase	Aspergillus oryzae	
Q4IIL7	Hypothetical protein	Gibberella zeae	
Q5AY15	Hypothetical protein	Emericella nidulans	
Q9WTT6	Guanine deaminase (EC 3.5.4.3)	Rattus norvegicus	guanine deaminase activity [IDA]
Q5RAV9	Hypothetical protein	Pongo pygmaeus	hydrolase activity [IEA]
Q4SCM3	Chromosome 12 SCAF14652, whole genome shotgun sequence. (Fragment)	Tetraodon nigroviridis	
Q502E8	Hypothetical protein zgc:112282	Brachydanio rerio	hydrolase activity [IEA]

Table S6: SCI-PHY helps to identify potential annotation errors. A portion of one subfamily of guanine deaminases from the PFAM amidohydrolase family (Amidohydro_1) is shown. Accession numbers in blue have experimental evidence of guanine deaminase activity. Accessions shown in red have been annotated with different functions and are potential misannotations. Accessions shown in green have no annotation (or only a very general GO annotation), and can be labeled as guanine deaminases. 17 sequences within this subfamily were labeled as guanine deaminases; these are not shown.
